# Double-parabolic-reflectors acoustic waveguides for high-power medical ultrasound

**DOI:** 10.1038/s41598-019-54916-2

**Published:** 2019-12-06

**Authors:** Kang Chen, Takasuke Irie, Takashi Iijima, Takeshi Morita

**Affiliations:** 1 Department of Human and Engineered Environmental Studies, The University of Tokyo, Chiba, 277-8563 Japan; 2 Microsonic Co., Ltd., Tokyo, 185-0012 Japan; 3 National Institute of Advanced Industrial Science and Technology (AIST), Tsukuba, 305-8569 Japan

**Keywords:** Acoustics, Biomedical engineering, Acoustics, Biomedical engineering

## Abstract

High intensity focused ultrasound therapeutics are widely used to noninvasively treat various types of primary tumors and metastasis. However, ultrasound penetration depth is shallowed with increasing frequency which limits the therapeutic accuracy for deep tissues. Although acoustic waveguides are commonly inserted into tissue for localized therapy, powerful ultrasound delivery is difficult. Here, we invent double-parabolic-reflectors acoustic waveguides, where high-power ultrasound emission and large mechanical vibration enhance the therapeutic efficiency. High-energy-density ultrasound with around 20 times amplification by two parabolic reflectors propagates through the thin waveguide between 1 to 2 MHz, and wideband large mechanical vibration at the waveguide tip from 1 kHz to 2.5 MHz accelerates the therapeutics. This fundamental work serves as a milestone for future biomedical applications, from therapeutics to diagnostics. Since the high-power ability at high frequencies, our waveguide will also open up new research fields in medical, bio, physics and so on.

## Introduction

Health of human beings is always the dominant concern in the modern society, which is continuously arousing further investments and development for medical devices. Among the medical equipment, ultrasound based medical devices have been universally recognized as the low-cost and human/environmental-friendly tool for diagnostics and therapeutics, applications mainly cover from finding the source of diseases (imaging of internal body structures)^1,2^ to treatments (tissue destruction, drug delivery, gene therapy, etc.)^3,4^. In typical ultrasound therapeutics, High Intensity Focused Ultrasound (HIFU) treatments with the frequency of 0.75 to 3 MHz and the focal intensity of over 1 kW/cm^2^ are utilized to noninvasively destruct the diseased tissue by acoustic focusing from the outside of human body. The cigar-shaped focal dimensions are usually in the order of 1–3 mm in the transverse direction and 8–15 mm in the beam axis direction. Such relatively large dimensions make HIFU transducers lose certain accuracy in localized therapy for deep tissues^5,6^ and some bioeffects including side burnings (by thermal-based therapies) and significant hemorrhage (by mechanical-based therapies)^7,8^ may be potentially induced. Alternatively, minimally invasive energy-based treatments (MIT) can be a promising candidate, it inserts the thin needle directly to the lesion to deliver different types of energy for treatments. MIT can limit the size of required incisions, localize the therapeutic region, and shorten the wound healing time, associated pain and risk of infection^9,10^. MIT possesses superior advantages for tumor therapeutics^11,12^ in liver, kidney, prostate, etc. However, conventional MIT including thermotherapy (radiofrequency ablation^13^, laser ablation^14^) and cryotherapy (cryoablation^15^) are intended to realize large-volume efficient therapy with over 4 mm in diameter and 5 mm in depth^3,16^, which limits the applications that require localized accurate therapy.

Although ultrasound-based MIT offers the opportunity for localized accurate therapy, the essential point is how to emit powerful ultrasound at the needle tip to increase the therapy efficiency. When arranging the energy source (usually piezoelectric element) at the needle tip that is similar to intravascular ultrasonic transducers (IVUS), the size is restricted which hinders the high-power generation^17–19^. Therefore, using acoustic waveguides to deliver powerful ultrasound from the outside of human body to the waveguide tip has received wide attention. However, the available designs are suffering from limited energy source (large diameter difference between piezoelectric element and waveguide)^20,21^ and energy loss during transmission (multiple changes of transmission mediums)^22^, so, high-power transmission had been impossible. The essence of these problems belongs to the difficult design of the transition portion between piezoelectric element and thin waveguides. To transmit more powerful ultrasound, although increasing contact surface area between piezoelectric element and waveguides were proposed^23–28^, high-power performances were still not satisfied due to the undesirable acoustic guidance of ultrasound in the waveguides (large reflections by the steep design of waveguides, etc.). Thus, no available acoustic waveguides were reported that can achieve high-power ultrasound propagation.

In this article we report a significant advancement in delivering high-power ultrasound in acoustic waveguides for efficient minimally invasive ultrasound therapeutics. By designing the 1^st^ parabolic reflector to focus ultrasound and the 2^nd^ parabolic reflector to guide ultrasound, powerful ultrasound with plane wavefront and enhanced energy density can be introduced into the thin waveguide for high-power transmission. Extremely large mechanical vibration at the waveguide tip can be realized between 1 kHz to 2.5 MHz, and large localized acoustic pressure can be emitted, which allow accurate and efficient therapeutics. For conventional HIFU transducers that focus ultrasound from the outside, the focusing performance strongly relies on the working medium, however, our waveguide can directly orient the thin waveguide tip to the lesion for treatments and the output performances can be conveniently controlled by the waveguide design. Due to the direct contact between the solid waveguide and tissue, additional large mechanical vibration for direct contact tissue damage^29–31^ can be achieved besides the heating effects. So, by combining these effects, the tissue destruction efficiency can be improved. This proposal will provide a new solution for accurate minimally invasive ultrasound therapeutics.

## Results

### Double-parabolic-reflectors acoustic waveguides

**Figure 1:**
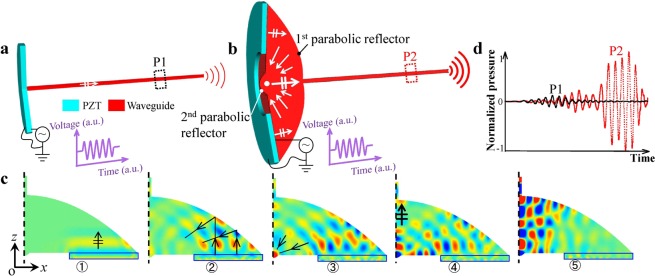
Principles of conventional waveguides and invented double-parabolic-reflectors waveguides. (**a**,**b**) Conceptual schematics of general configuration of piezoelectric element (PZT) and waveguide (**a**) and proposed configuration with parabolic-focusing waveguides (**b**). In (**a**) PZT is attached to a thin waveguide with large diameter difference. In (**b**) PZT ring is attached to a large-end-surface waveguide with double parabolic reflectors to focus and guide ultrasound. (**c**) Working steps of double-parabolic-reflectors waveguides. Vibration amplitude of the incident plane-wavefront ultrasound is enhanced by double parabolic reflectors, color represents the vibration velocity in *z* direction. (**d**) Transmitted ultrasound comparison for two configurations. Simulated acoustic pressure at positions P1 and P2 that is 200 mm away from the left end of thin waveguides under same voltage and 1.4046 MHz when thin waveguides are *ϕ* 1 mm in diameter and PZT is attached to backing layers^32^.

In typical thin waveguides as shown in Fig. 1a, the piezoelectric element (PZT) works in thickness mode vibration and the emitted weak ultrasound is propagating through the thin waveguides. Waveguides are required to be thinner than several mm for minimally invasive treatments^23–28^, therefore, large diameter difference exists between PZT and waveguides, which limits the amount of energy being directly transmitted to the waveguides. To enhance the energy input, we invented double-parabolic-reflectors acoustic waveguides as shown in Fig. 1b. Two important features should be highlighted. First, a large-size PZT ring can be attached to the large-end-surface waveguide, which indicates that more energy can be directly introduced to the waveguide. Second, plane-wavefront ultrasound with enhanced vibration amplitude can be achieved by double parabolic reflections. The first parabolic reflector is used to increase the contact surface area between PZT and waveguides and to focus ultrasound. The second parabolic reflector is to transfer the focused ultrasound to an enhanced plane-wavefront ultrasound. Two parabolic reflectors share the same focal point and the resulted ultrasound is guided to the thin waveguides for high-power transmission. The working principle can be described by five steps as shown in Fig. 1c. In the beginning, a plane-wavefront ultrasound is emitted by PZT and propagated into the waveguide. Then, the plane-wavefront ultrasound is reflected by the 1^st^ parabolic reflector. The reflected ultrasound is focused to the focal area. In the next, the focused ultrasound is reflected by the 2^nd^ parabolic reflector and transferred to a plane-wavefront ultrasound with larger energy density. Eventually, the enhanced plane-wavefront ultrasound is propagating through the thin waveguide. Theoretically speaking, we have proved that the proposed acoustic waveguide can generate powerful ultrasound that is over one order-of-magnitude of conventional configuration in acoustic pressure and over two orders-of-magnitude in power^32^. In Fig. 1d, we also illustrate the comparison of acoustic pressure in *ϕ* 1 mm waveguides under 1.4046 MHz. It is obvious that the invented waveguide possesses superior performance in generating powerful ultrasound.

### Transducer design and characterization

**Figure 2:**
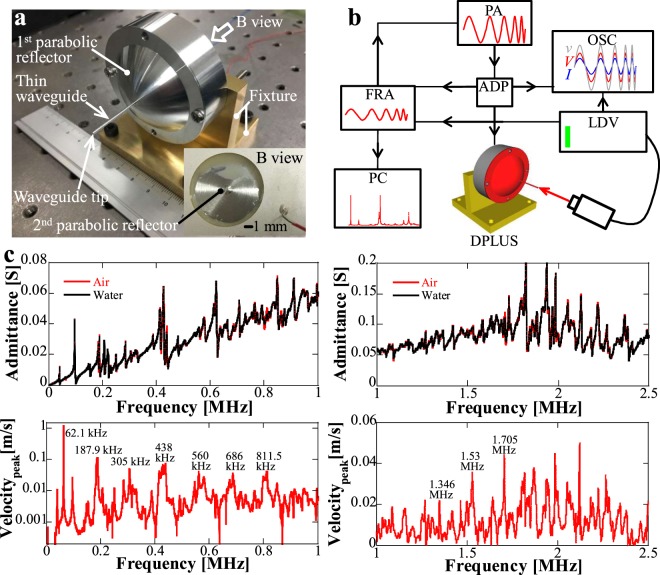
Device architecture and experimental schematic. (**a**) Photograph of the manufactured Double Parabolic refLectors wave-guided Ultrasonic tranSducer (DPLUS). The core component of DPLUS is the double-parabolic-reflectors acoustic waveguide. The thin waveguide is *ϕ* 1 mm in diameter and the focal lengths of the 1^st^ and 2^nd^ parabolic reflectors are 10 mm and 0.5 mm. The PZT ring is 20 mm in outer radius, 8 mm in inner radius, and 1.1 mm in thickness. (**b**) Experimental setup used to characterize the frequency responses of DPLUS. PC personal computer, FRA frequency response analyzer, PA power amplifier, ADP adaptor, OSC oscilloscope, LDV laser doppler vibrometer. (**c**) Measured frequency responses of DPLUS in terms of admittance and vibration velocity. In admittance curves, water represents that admittance of DPLUS was measured when the waveguide tip was immersed into water. The vibration velocity was obtained along the longitudinal direction of thin waveguide under 1 V_pp_.

The essential theory in solid waveguides is the propagation modes. And dispersion phenomenon is the reason why waves with different wavelengths can propagate at different speeds in each propagation mode in the same material. In cylindrical mechanical waveguides for minimally invasive treatments, axially symmetrical “longitudinal” waves are most commonly selected^20–28^, the characteristic equation of Pochhammer-Chree wave is given by^33^

$${k}^{2}{k}_{{\rm{t}}}\frac{{J}_{0}({k}_{{\rm{t}}}a)}{{J}_{1}({k}_{{\rm{t}}}a)}+[-\frac{1}{2}{(\frac{\omega }{{c}_{{\rm{t}}}})}^{2}]\frac{1}{a}+{[\frac{1}{2}{(\frac{\omega }{{c}_{{\rm{t}}}})}^{2}-{k}^{2}]}^{2}\frac{{J}_{0}({k}_{{\rm{d}}}a)}{{J}_{1}({k}_{{\rm{d}}}a){k}_{{\rm{d}}}}=0$$

where ${k}_{{\rm{d}}}=\sqrt{{(\omega /{c}_{{\rm{d}}})}^{2}-{k}_{0}^{2}},\,{k}_{{\rm{t}}}=\sqrt{{(\omega /{c}_{{\rm{t}}})}^{2}-{k}_{0}^{2}}$, ${c}_{{\rm{d}}}=\sqrt{(\lambda +2\mu )/\rho },\,{c}_{{\rm{t}}}=\sqrt{\mu /\rho }$*, ω* and *k* are the angular frequency and wavenumber of the propagating wave, *J*_0_(*x*) and *J*_1_(*x*) are the Bessel functions of the first kind of order zero and one*, λ* and *μ* are Lame’s constants, and *a* is the radius of the waveguide. Depending on the modes, the propagation speeds are different and the vibration shapes at the cross section of the waveguides are complicated with increasing number of the modes. Here, the first mode was selected (see Supplementary Fig. 1), and the diameter of the thin waveguide was determined as 1 mm with 20 mm long considering the fabrication process. In terms of two parabolic reflectors, amplification ratio of the vibration amplitude of the incident ultrasound is the key design purpose. The carried energy *E* of a plane-wavefront ultrasound averaged over a wavelength is related to $\frac{1}{\lambda }{\int }_{V}\rho {(\frac{\partial u}{\partial x})}^{2}dV=\rho {A}^{2}{\omega }^{2}S$, where *u* is the vibration displacement, *V* is the volume of the wave, *A* is the vibration amplitude*, ρ* is the density of the waveguide material, and *S* represents the area of the wavefront^33^. If the mode conversion^34^ during parabolic reflections to cause loss of energy carried by longitudinal vibration is neglected, the amplification ratio of the vibration amplitude equals to $\sqrt{({r}_{2}^{2}-{r}_{1}^{2})/4{m}^{2}}$ (see Supplementary Note 1 for the detailed derivation), whe*r*e *r*_1_ and *r*_2_ represent the inner and outer radius of the PZT ring, *m* is the focal length of the 2^nd^ parabolic reflector. In the current optimized design, *r*_1_ and *r*_2_ were 8 mm and 20 mm, respectively, PZT thickness was 1.1 mm, *m* equaled to 0.5 mm, and the focal length of the 1^st^ parabolic reflector was 10 mm, therefore, the amplification ratio reaches to around 18.3. A prototype named Double Parabolic refLectors wave-guided Ultrasonic tranSducer (DPLUS) was fabricated as shown in Fig. 2a, the core component is the acoustic waveguide with double parabolic reflectors. The waveguide was made of duralumin, the fixtures were made of brass, and a soft-type PZT with mechanical quality factor *Q*_m_ of around 75 was used.

To examine the capability of minimally invasive tissue treatments by direct-contact mechanical vibration and thermal destruction, we characterized the output performances of DPLUS based on vibration velocity and emitted acoustic pressure. For low frequency high intensity ultrasound ablation using waveguides, direct contact ablation, cavitation, acoustic streaming and pressure wave components are the main effects, and they are directly related to vibration velocity at the waveguide tip and frequency^35,36^. Therefore, vibration velocity is regarded as one of the important evaluation indexes. For HIFU ablation, the working mechanisms of thermal and mechanical destruction are associated with frequency, acoustic pressure, and cavitation^7,8^. Thus, acoustic pressure is considered as an evaluation index for high frequencies.

To characterize the mechanical vibration at the waveguide tip, frequency responses of DPLUS in terms of admittance and vibration velocity were investigated using the experimental setup shown in Fig. 2b. The vibration velocity was measured along the longitudinal direction of the thin waveguide under 1 V_pp_ and the results are shown in Fig. 2c (see Supplementary Fig. 2 for the lateral vibration velocities). There are three important findings. First, the admittance curve was flattened compared with admittance from a single PZT ring without attaching to the waveguide (see Supplementary Fig. 3 for the admittance curve of PZT ring), meanwhile, more admittance peaks appeared. As a result, there are wide frequency ranges showing large vibration velocities. Second, frequencies with large mechanical vibration exactly correspond to the frequencies with obvious admittance change (see Supplementary Fig. 4 for the detailed admittance change compared to simulation). Third, from the vibration velocity curves, large mechanical vibration can be constantly observed from 0 to 2.5 MHz, which offers flexible selection of frequencies with large vibration for effective tissue destruction. At low frequencies of 62.1, 187.9, 305.1, and 438.1 kHz, velocity peaks of 1.22, 0.114, 0.05, and 0.075 m/s can be achieved, more importantly, dense and large vibration velocity peaks occur at megahertz frequencies, vibration velocity at 0.811, 1.346, 1.53 and 1.705 MHz can be over 41.5, 22.3, 35.8, and 44.18 mm/s, respectively. Compared with conventional kilohertz range high-power Langevin transducers^29–31^, these results were regarded as a breakthrough in two aspects: providing wideband frequencies with large vibration amplitudes and extending high mechanical vibration to megahertz frequency range.

**Figure 3:**
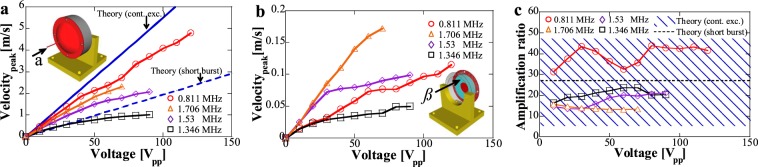
Vibration velocity characteristics under continuous excitation. Vibration velocity at the (**a**) waveguide tip, (**b**) PZT surface, and (**c**) the calculated amplification ratio under continuous excitation. The theoretical curves in (**a**,**c**) were provided by simulation using PZFlex (see Supplementary Fig. 6 for the simulation model). In (**a**,**b**), *α* direction represents the longitudinal direction along the thin waveguide and *β* direction is the thickness direction of the PZT ring. In (**c**) the amplification ratio was calculated by the vibration velocity of waveguide tip divided by the vibration velocity at the PZT surface under the same input.

**Figure 4:**
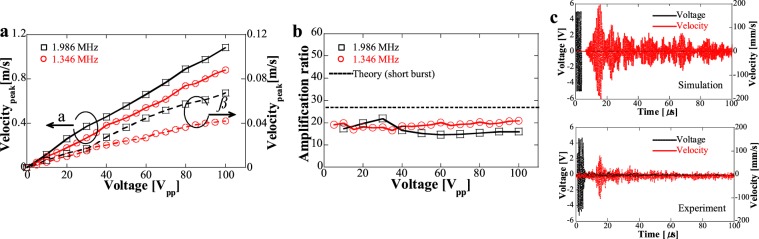
Vibration velocity characteristics under short burst excitation. (**a**) Vibration velocity at the waveguide tip, PZT surface and (**b**) calculated amplification ratio under 5-cycles short burst excitation. In (**c**) the waveform at the waveguide tip under 10 V_pp_ excitation is compared between simulation and experiment.

**Figure 5:**
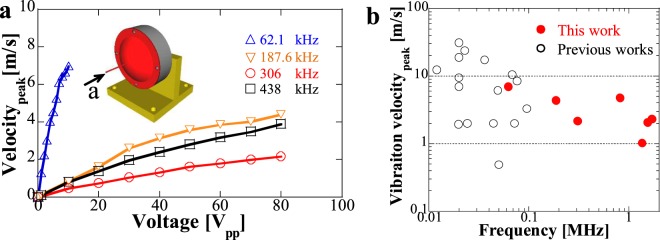
Vibration velocity at low frequencies and performance comparison of mechanical vibration. (**a**) Measured vibration velocity at low frequencies under continuous excitation. (**b**) Vibration velocity comparison with conventional transducers. In conventional transducers, long horn or long waveguides were typically used for tissue ablation. Citations to previous work are provided in the Supplementary Fig. 7.

For further exploration of vibration velocity characteristics, the aforementioned frequencies were typically selected, and measurements under continuous and 5-cycles short burst excitation were shown in Figs. 3–5 (see Supplementary Fig. 5 and Note 2 for the designed impedance matching circuit for vibration velocity measurements). Under continuous excitation as shown in Fig. 3, vibration velocity at the waveguide tip (*α* direction) and PZT surface (*β* direction) were measured, and an amplification ratio *R*_A_ defined by the vibration velocity at the waveguide tip divided by PZT surface vibration velocity at the same input was calculated. In Fig. 3a, with increasing exciting voltage, vibration velocity at the waveguide tip showed an initial linear increase and a subsequent saturation for 1.346 and 1.53 MHz, in comparison, a continuous increase for 0.811 and 1.705 MHz can be observed. Theoretical curves were provided by numerical simulation at 1.29 MHz under continuous and 5-cycles burst excitation, which delineated the linear relationship due to the underestimation of heat generation and associated nonlinearity phenomena^37^. It is important to highlight that at megahertz frequencies, DPLUS can realize extremely large vibration velocity at the waveguide tip, 4.8, 1.015, 2.08, and 2.315 m/s peak value of vibration velocity can be achieved at around 0.807, 1.3454, 1.53 and 1.702 MHz under the exciting voltage of 120, 90, 90 and 70 V_pp_, respectively. These results proved the high mechanical vibration of our DPLUS at megahertz range. Regarding the vibration velocity along the thickness direction at the PZT surface as shown in Fig. 3b, with increasing voltage, 0.811 and 1.706 MHz indicated a more linear relationship while the other frequencies showed a gradual saturation. The amplification ratio *R*_A_ was calculated as shown in Fig. 3c, theoretical results under continuous excitation were indicated by an area due to the uneven distribution of vibration velocity at the PZT surface. In experiments, *R*_A_ of 0.811 MHz (around 40) possessed a large discrepancy with *R*_A_ of other frequencies (around 20), which indicates that 1.346, 1.53 and 1.706 MHz are closer to the thickness mode vibration of PZT under continuous excitation.

Under 5-cycles short burst excitation, vibration velocity at the waveguide tip and PZT surface were measured under 1.346 and 1.986 MHz as shown in Fig. 4. From Fig. 4a, one can observe that vibration velocities delineated a linear relationship with increasing voltage and showed the peak values of 0.88 (at 1.346 MHz) and 1.08 (at 1.986 MHz) m/s under 100 V_pp_. The amplification ratios in Fig. 4b indicated similar values between experiment and simulation, 27 and 20 times of amplification ratios were achieved in simulation and experiments. In our previous calculation, the amplification ratio should be around 18.3 without considering the mode conversion loss and resonance of the structure, the difference indicates that resonance assists to enhance the amplification ratio under short burst excitation. It is significantly important to notice that large amplification ratio can be achieved for the designed DPLUS. Compared with the focusing techniques in HIFU transducers^38,39^ (commonly with the focusing gain of several to a hundred times) and conventional Langevin transducers with horn deign^40^ (commonly with amplification lower than 10), our waveguide showed promising focusing performance. It also needs to be noticed that if we design additional focusing part at the thin waveguide tip such as focusing lens, the focusing performance can be further improved. Furthermore, transient vibration velocity shown in Fig. 4c compared the simulation and experimental results under 10 V_pp_, 1.29 and 1.346 MHz, respectively. It can be found that very similar waveforms can be obtained at the waveguide tip despite of the difference in vibration velocity values, experimental results were around half of simulation results. In summary, from the mechanical vibration characteristics at high frequencies, we showed that the invented acoustic waveguide successfully achieved extremely large mechanical vibration with high amplification ratio.

At low frequencies, the mechanical vibration was measured under continuous excitation as shown in Fig. 5a. Different from high frequencies where double-parabolic-reflectors structure serves as the focusing mechanism by double parabolic reflections, for ultrasound at low frequencies with large wavelength, the 1^st^ parabolic reflector works as a horn structure in Langevin transducers to increase the vibration amplitude under resonance. In other words, the large vibration amplitude is due to the small diameter of the thin waveguide. It can be observed that 62.1 kHz offers the largest potential to increase the vibration velocity, 7 m/s at 10 V_pp_ can be easily achieved. Other frequencies showed smooth increase of vibration velocity, around 4.4, 2.2, and 3.9 m/s can be realized under 80 V_pp_ at 187.6, 306, and 438 kHz, respectively. Comparison with conventional transducers using long horn or waveguides for tissue ablation was shown in Fig. 5b, there are two important aspects to be emphasized for the designed DPLUS. First, from the perspective of vibration velocity, the proposed acoustic waveguide can achieve similar level of vibration velocity at frequencies below 100 kHz which enables tissue ablation at low frequencies by direct-contact mechanical vibration. Second, from the view of working frequency, we can extend it to megahertz range while keeping large vibration velocity. It is not available for conventional types because large vibration displacement that can be easily achieved at low frequencies was considered to be effective to increase the ablation area^41,42^ and high-frequency transducers that can realize large mechanical vibration is difficult to design. For our DPLUS working at megahertz ranges, in addition to tissue ablation by thermal effects, the solid waveguide with large mechanical vibration can directly contact tissue so that direct-contact mechanical ablation can be induced, by combining these two effects, the therapeutic efficiency can be enhanced.

**Figure 6:**
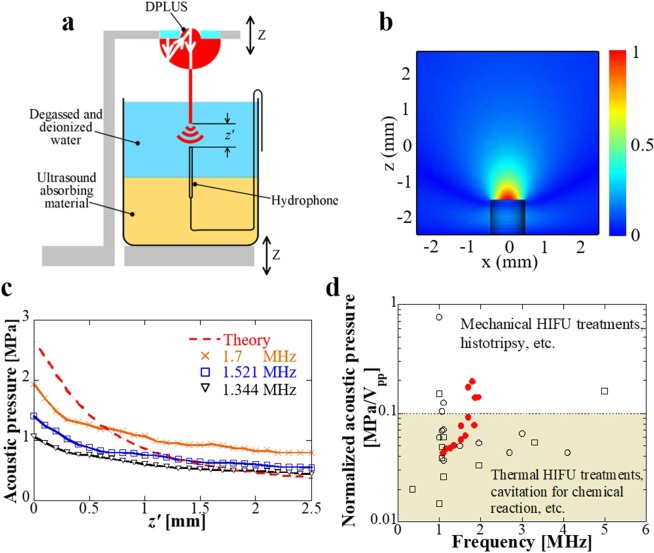
Evaluation of localized powerful ultrasound emission in water. (**a**) Illustration of axial acoustic pressure measurement. (**b**) Simulated acoustic pressure distribution at the waveguide tip under 1.164 MHz. (**c**) Measured axial acoustic pressure under 10 V_pp_. (**d**) Acoustic pressure comparison with conventional HIFU transducers. Normalized acoustic pressure is evaluated as the acoustic pressure divided by the input voltage. Black circles represent the peak positive pressure, black squares indicate the peak negative pressure. The superior performance of DPLUS (red dots) is comparable to transducers for histotripsy. Citations to previous work are provided in the Supplementary Fig. 8.

Besides mechanical vibration characteristics, important parameters related to thermal treatments were also explored. Here, we investigated the emission of localized powerful ultrasound in water when the waveguide tip was immersed into water as shown in Fig. 6a. The distribution of acoustic pressure in water was extracted from simulation at 1.164 MHz as indicated in Fig. 6b, one can observe that large acoustic pressure can be generated near the waveguide tip and the area is constrained to 1 mm × 1 mm range. If we design active/passive acoustic focus at the waveguide tip, focalized large acoustic pressure can be realized. The axial acoustic pressure at different frequencies were measured as shown in Fig. 6c. Experimental results were smaller than simulation near the waveguide tip and one possible reason is that experiments were influenced by the spatial averaging effects. In simulation, acoustic pressure close to the waveguide tip was 2.65 MPa under 10 V_pp_. And the acoustic pressure was dramatically reduced to be around 1/2 and 1/4 of the acoustic pressure near the waveguide tip when *z*′ was around 0.55 mm and 1.35 mm. In experiments, 1.344, 1.521, and 1.7 MHz offer around 1, 1.2, and 2 MPa of maximum acoustic pressure, and the acoustic pressure was reduced to be around 1/2 when *z*′ was around 1.4 mm. These results support the generation of localized ultrasound. Furthermore, the output acoustic pressure was compared with conventional HIFU transducers for ultrasound therapeutics as shown in Fig. 6d, normalized acoustic pressure was evaluated as the acoustic pressure divided by the input voltage. For our waveguide, the measured acoustic pressure was at 10 V_pp_ but the compared normalized pressure was calculated to the maximum applied voltage according to the vibration velocity values shown in Fig. 3a. In comparison, it can be found that for mechanical HIFU treatments such as histotripsy, transducers are required to have a high normalized acoustic pressure of over 0.1 MPa/V_pp_ while transducers for typical thermal treatments need a moderate value from 0.01 to 0.1 MPa/V_pp_. Between 1 to 2 MHz, our waveguide possesses a good performance and can be comparable to transducers for histotripsy applications although it is difficult to achieve as high acoustic pressure as those transducers (usually over 80 MPa peak positive pressure and 20 MPa peak negative pressure are required for histotripsy applications). In conclusion, our waveguide shows the ability to realize large and localized acoustic pressure at the waveguide tip, which is promising for practical treatments.

### Applications of the double-parabolic-reflectors acoustic waveguides for tissue ablation

**Figure 7:**
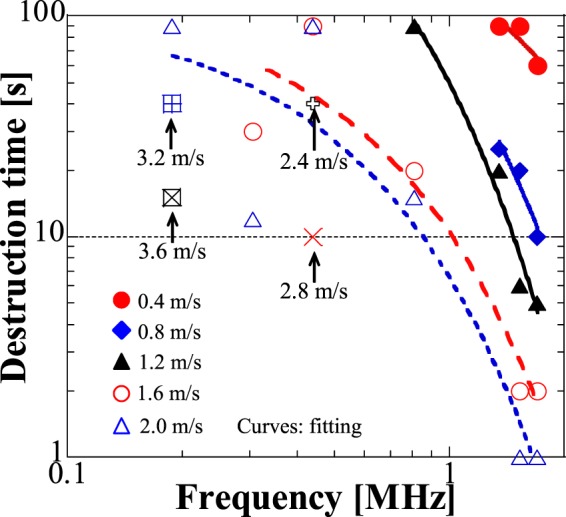
Tissue destruction abilities at different frequencies. Commercial animal fat was attached to the thin waveguide tip and it was completely turned to fluid statement after applying ultrasound, the destruction time refers to the required time for turning the fat to fluid statement and the measurements were conducted at the same vibration velocities (at free condition without attaching to loads) for different frequencies.

To prove our invented acoustic waveguides with double parabolic reflectors for effective therapeutics by combining large mechanical vibration induced direct-contact mechanical ablation and large localized acoustic pressure induced thermal ablation, here we present the basic results of soft tissue destruction. Soft tissue of commercial animal fat was attached to the thin waveguide tip, the consumed time to destruct the fat to entirely fluid condition was recorded under different frequencies and vibration velocities, results are shown in Fig. 7. Under the same free vibration velocity at the waveguide tip, frequencies from 1 to 2 MHz dramatically enhanced the destruction efficiency, and the destruction time was reduced by around two magnitudes when the free vibration velocities were larger than 1.6 m/s. A comparison of fat destruction at low and high frequencies can be found in Supplementary Movie 1 and Movie 2. At each frequency, increasing mechanical vibration saved the destruction time to a different extent, 0.1 to 1 MHz ranges show smooth decrease of destruction time and 1 to 2 MHz ranges indicate the sharp reduction of destruction time. When the working frequency is relatively low (<0.5 MHz), the thermal effects for tissue destruction are not strong so that direct-contact mechanical effects play an important role. In comparison, at high frequencies (1 to 2 MHz), ultrasound can be easily absorbed by soft tissue so that both thermal and mechanical effects are effective for tissue destruction. Therefore, by combining these two effects, efficient tissue destruction can be achieved. In addition, since the waveguide can directly contact with tissue and achieve large mechanical vibration, solid tissue destruction is promising in the future. In conclusion, to effectively destruct tissue, high vibration amplitudes at low frequencies (0.1 to 1 MHz) are required, however, at high frequencies (1 to 2 MHz), the ablation efficiency is high even at low vibration amplitudes due to the combination of thermal effects and mechanical effects.

## Discussion

Our work demonstrates the effectiveness of double parabolic reflectors to enhance the energy density of the incident ultrasound and to transmit powerful ultrasound. Combining experimental and theoretical approaches, we found the acoustic waveguide can not only deliver localized powerful ultrasound at the waveguide tip for ultrasound thermal therapeutics but also generate wideband large mechanical vibration for direct-contact mechanical tissue ablation, therefore, the therapeutic efficiency is high. Compared with conventional low-frequency (<100 kHz) high-power transducers for tissue destruction, our technique extends the working frequency to 2.5 MHz while keeping high mechanical vibration. Besides, compared with typical HIFU transducers for common thermal and mechanical ablation, our waveguide can realize similar output performance while speeding up the therapeutic by direct-contact mechanical effects. These new introduced features of our waveguide are of great importance to examine and explore some basic physics in the research field. For further investigation of our acoustic waveguides, first, matching sections at the thin waveguide tip can be added to ease the large acoustic impedance mismatch. Second, coating to the waveguide surface seems to be promising to avoid energy loss^22^. Third, long waveguide that can get access to deep tissues will be considered for future development. Fourth, high frequency acoustic waveguides larger than 10 MHz for treatments will be a breakthrough for ultrasound therapeutics. By realizing these future works, performances of our waveguide can be further improved, and many biomedical applications can be opened. As for potential applications, in addition to minimally invasive ultrasound therapeutics, our waveguide can also be utilized for ultrasound imaging of large tissue (liver, etc.), ultrasound microscope^28^, cavitation for chemical reaction^43–45^, acoustic tweezer for cell manipulation^46^, drug delivery^4,47^, gene therapy^47^, etc. In conclusion, in this fundamental work, we introduced new features to our waveguide that are not possible for conventional transducers. As a result, many important biomedical applications might become possible. Therefore, this work is regarded as a milestone in achieving important biomedical applications in the future.

## Methods

### Measurement setup

To characterize the vibration velocity under single frequency excitation, continuous/burst RF signals generated by a waveform generator (33250 A, Keysight Technologies) and amplified by a power amplifier (T145-5027D, THAMWAY CO., LTD) were applied to DPLUS. Impedance matching circuit shown in Supplementary Fig. 5 and Note 2 was designed to supply enough power to DPLUS. The vibration velocity signals were measured by a Laser Doppler Vibrometer (LV-1800, ONO SOKKI Co., Ltd.). When the thin waveguide tip was immersed into water for acoustic pressure measurements, a needle type hydrophone (HY05N, Toray Engineering) with the tip effective diameter of 0.5 mm was placed concentrically below the thin waveguide tip. For tissue destruction experiments, commercial animal fat from cow with the volume of 72 mm^3^ was added to the waveguide tip each time, and the destruction of fat was recorded by a microscope (HOZAN TOOL IND.CO., LTD.).

### Simulation

The finite element software Femtet (Murata Software Co., Ltd.) and PZFlex (Weildlinger Associates, Los Altos, CA) were used in simulation. Simulation models are shown in Supplementary Fig. 6. Femtet was utilized to obtain the admittance characteristics and PZFlex was to get the transient vibration velocities and acoustic pressure distribution. Simulation models were built as axisymmetric models to improve the calculation efficiency. PZT polarized in thickness direction was selected as a soft type PZT C-62 from Fuji Ceramics Co., Ltd. with thickness of 1.1 mm and inner/outer radius of 8/20 mm, waveguide was made of duralumin with the density of 2790 kg/m^3^, bulk velocity of 6420 m/s and poison’s ratio of 0.3467, and the water medium possessed the sound velocity of 1496 m/s. The waveguide radius $a$ was 0.5 mm, the focal length of the 1^st^ parabolic reflector *n* and the 2^nd^ parabolic reflector *m* equaled to 10 and 0.5 mm. In PZFlex simulation, the excitation frequency to obtain the vibration velocity characteristics was 1.29 MHz and the frequency for acoustic pressure extraction was 1.164 MHz. To reach the stable output fields, the element size for meshing in PZFlex was 50 µm and the total simulation time was 1 ms.

## Supplementary information

Supplementary Information

Supplementary Information movie1

Supplementary Information movie2
