# Supplementary material for: Double-parabolic-reflectors acoustic waveguides for high-power medical ultrasound
Source: Sci Rep. 2019 Dec 6;9:18493. doi: 10.1038/s41598-019-54916-2 (PMC6898714; doi:10.1038/s41598-019-54916-2)
Supplement: Supplementary file 1 — Supplementary Information [file 41598_2019_54916_MOESM1_ESM.docx]

Supplementary information for:

Double-parabolic-reflectors acoustic waveguides for high-power medical ultrasound

Kang Chen, Takasuke Irie, Takashi Iijima, & Takeshi Morita


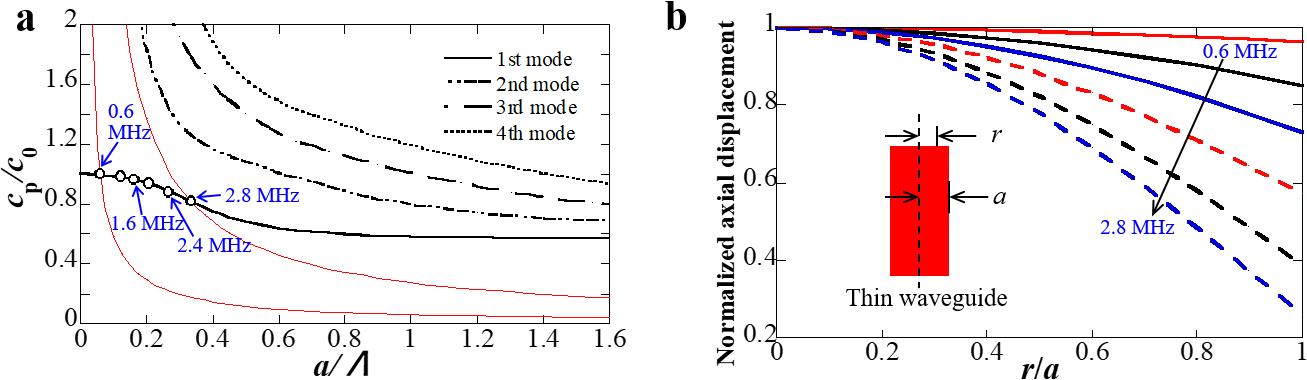


**Supplementary Figure 1: Calculated propagation mode parameters by solving the Pochhammer-Chree wave equation.** (a) Selection of the 1^st^ mode of the Pochhammer-Chree wave and (b) axial displacement distribution at the cross section of the thin waveguide under different frequencies (equivalent to different $a/\Lambda$, where $\Lambda$ is the wavelength of the propagating wave). Each calculated curve in b is normalized so that the maximum is 1. The axial displacement is uneven especially under high frequencies.

**Supplementary Note 1: Amplification ratio by double parabolic reflectors**

We divided the ultrasound propagation in the waveguides into three regions as shown in Supplementary Figure 6c. Region $I$ is between two parabolic reflectors where ultrasound is focused and guided, region $\Pi$ is the cylindrical portion of the waveguide where ultrasound with specific propagation modes is propagating through, region $III$ is from the waveguide tip to the water medium where ultrasound is emitted from the waveguide tip. To analyze the amplification ratio by double parabolic reflectors, we focused on region $I$.

In region$I$, two parabolic reflectors are the key structures to focus and guide ultrasound. The 1^st^ and 2^nd^ parabolic reflectors are written as:

|  | $p_{1}:z=\frac{-r^{2}}{4n}+n, (a\leq r\leq2n)$ | (1) |
| --- | --- | --- |
|  | $p_{2}:z=\frac{r^{2}}{4m}-m, (0\leq r\leq2m)$ | (2) |

where *n* and *m* are the focal length of the 1^st^ and 2^nd^ parabolic reflector, $a$ is the radius of the thin waveguide. It is worthwhile to mention that plane incidence will be uniquely focused to the focal point at the same time for parabolic reflectors, and vice versa. Since the incident ultrasound $u_{1}$ can be regarded as a plane-wavefront incident ultrasound, $u_{1}$ can be expressed by Supplementary Equation (3). And after reflections by two parabolic reflectors, a plane-wavefront ultrasound $u_{2}$ with enhanced vibration amplitude is generated, which can be formulated by Supplementary Equation (4).

|  | $u_{1}\left( z,t \right)=A_{1}e^{i(\omega t-k_{0}z)}, \left( r_{1}\leq r\leq r_{2}, t\to0 \right)$ | (3) |
| --- | --- | --- |
|  | $u_{2}\left( z,t \right)=A_{2}e^{i\left( \omega t-k_{0}z \right)},\left( 0\leq r\leq2m \right).$ | (4) |

In Supplementary Equations (3) and (4), $A_{1}$ and $A_{2}$ are the vibration displacement amplitudes of $u_{1}$ and $u_{2}$, $\omega$ is the angular frequency, and $k_{0}$ is the wavenumber of the propagating wave in region$I$. During reflections by parabolic reflectors, energy carried by longitudinal waves will be partially transferred to the energy carried by transverse waves due to mode conversion, energy loss of longitudinal wave needs to be considered:

|  | $\beta=\frac{E_{2}}{E_{1}}$ | (5) |
| --- | --- | --- |

where $E_{1}$ and $E_{2}$ are the energy carried by longitudinal waves before and after reflections. The carried energy $E_{i}$ of a plane-wavefront ultrasound averaged over a wavelength is related to $\frac{1}{\lambda}\int_{V} \rho\left( \frac{\partial u}{\partial x} \right)^{2}dV=\rho A_{i}^{2}\omega^{2}S_{i}$, where $i=1,2$, $\rho$ is the material density of the waveguide, $S_{1}$ and $S_{2}$ represent the area of the wavefront of $u_{1}$ and $u_{2}$. Therefore, by replacing $E_{1}$ and $E_{2}$ in Supplementary Equation (5), we can obtain:

|  | $\frac{A_{2}}{A_{1}}=\sqrt{\beta\frac{S_{1}}{S_{2}}}.$ | (6) |
| --- | --- | --- |

From Supplementary Equation (6), it can be found that enhancement of the vibration displacement amplitude of the resulted wave depends on $\sqrt{{S_{1}}/{S_{2}}}$. $\sqrt{{S_{1}}/{S_{2}}}$ can be formulated by:

|  | $\sqrt{\frac{S_{1}}{S_{2}}}=\sqrt{\frac{\pi(r_{2}^{2}-r_{1}^{2})}{\pi\left( 2m \right)^{2}}}=\sqrt{\frac{r_{2}^{2}-r_{1}^{2}}{4m^{2}}}.$ | (7) |
| --- | --- | --- |

where $r_{1}$ and $r_{2}$ represent the inner and outer radius of the PZT ring. It is obvious that enhancement of the ultrasound can be adjusted by the dimension of PZT ring and the 2^nd^ parabolic reflector. In the current optimized design, outer radius of PZT ring $r_{2}$ is designed to be equal to $2n$.


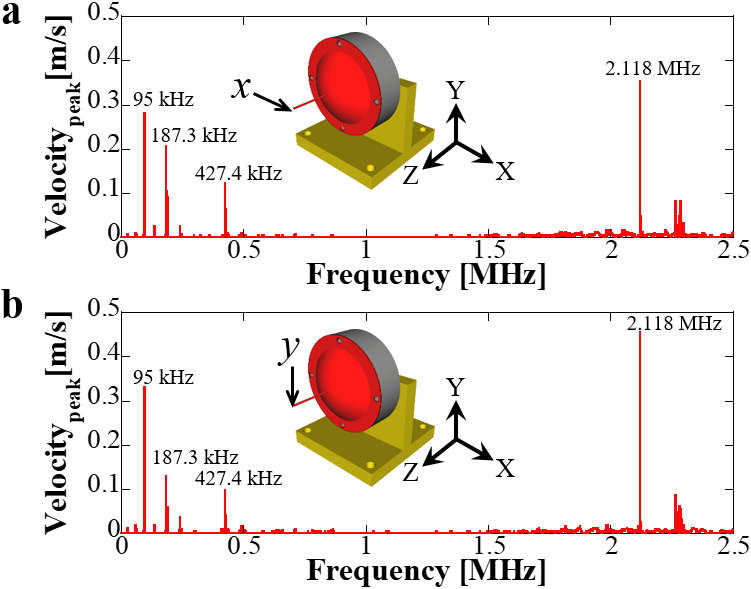


**Supplementary Figure 2: Measured lateral vibration velocities**. Lateral vibrations under 1 V_pp_ in (a) *x* and (b) *y* directions. Results showed the velocity peaks at low frequencies (95, 187.3, and 427.4 kHz) and high frequencies (2.118 MHz) with very small vibration between 0.5 to 2 MHz.

**Supplementary Figure 3: Measured admittance curve of PZT ring.** The PZT ring is polarized in the thickness direction with 1.1 mm in thickness. The largest admittance peak corresponds to the thickness mode of PZT ring.


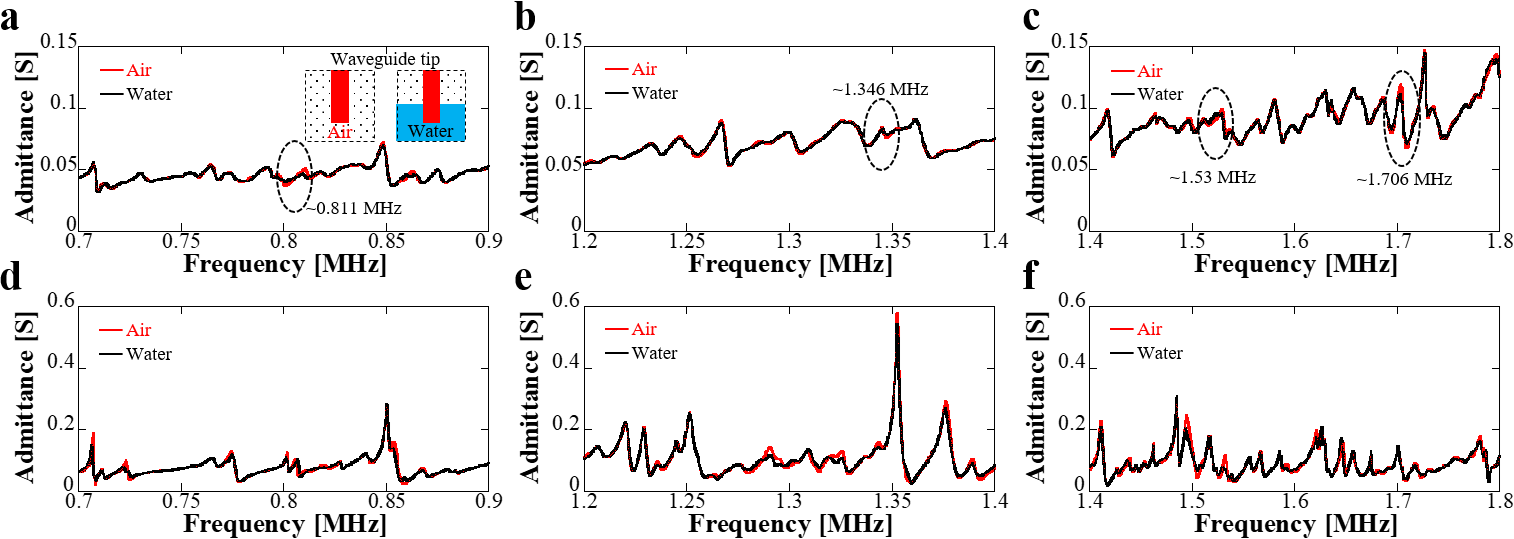


**Supplementary Figure 4: Detailed admittance curves comparison.** Admittance curves by (a)-(c) experiments and (d)-(f) simulation. First, similar tendency of admittance can be observed between experiments and simulation. Second, when the waveguide tip is immersed into water, large admittance changes can be observed at certain frequencies. At these frequencies, large mechanical vibration can be obtained.


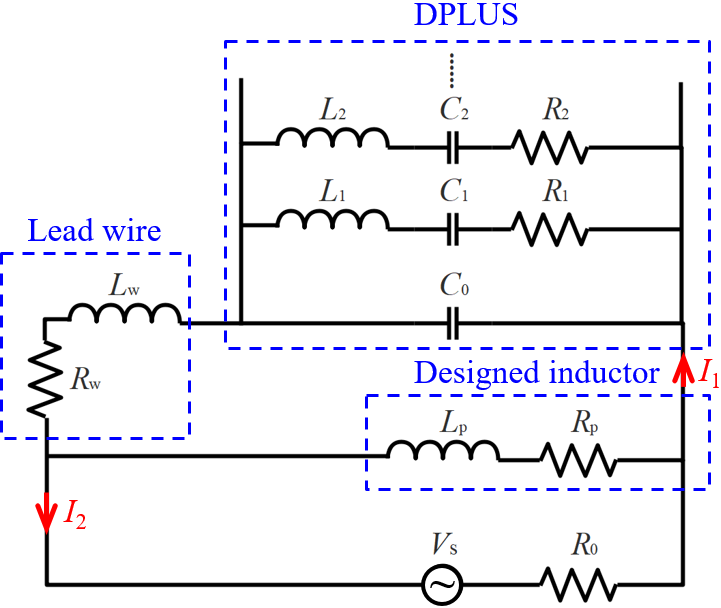


**Supplementary Figure 5: Circuit design for impedance matching.** Designed inductor is modelled as an inductor *L*_p_ and a resistor *R*_p_, *L*_w_ and *R*_w_ are the inductance and resistance of lead wire that directly connected to DPLUS for power supply, *R*_0_ is the output impedance of power amplifier, DPLUS is modelled by the equivalent circuit. The design purpose was to supply enough power to DPLUS. Supplementary Note 2 shows the detailed calculation of the designed inductor.

**Supplementary Note 2: Design of inductors for impedance matching**

If directly connect DPLUS to the power amplifier, maximum power supply is not possible due to the impedance mismatch. In other words, low impedance of DPLUS results in high current so that can exceed the threshold of power amplifier without reaching the maximum power output. Therefore, impedance matching circuit design is an important process to drive DPLUS. Here, we designed an inductor parallelly connected to DPLUS which shows good matching performance. The design purpose is to reduce the current *I*_2_ shown in Supplementary Fig.5 and supply enough power to DPLUS for the selected frequencies (0.811, 1.346, 1.53, 1.706 MHz). To this objective, we calculate the impedance *Z* of the whole device (the LCR branches of DPLUS are neglected):

| $Z=\frac{1}{\frac{1}{j\omega L_{p}+R_{p}}+\frac{1}{j\omega L_{w}+R_{w}+\frac{1}{j\omega C_{0}}}}=\frac{1}{\frac{R_{p}-j\omega L_{p}}{R_{p}^{2}+\omega^{2}L_{p}^{2}}+\frac{R_{w}-j\left( \omega L_{w}-\frac{1}{\omega C_{0}} \right)}{R_{w}^{2}+\left( \omega L_{w}-\frac{1}{\omega C_{0}} \right)^{2}}}=$ |  |
| --- | --- |
| $\frac{\left( R_{p}^{2}+\omega^{2}L_{p}^{2} \right)\left[ R_{w}^{2}+\left( \omega L_{w}-\frac{1}{\omega C_{0}} \right)^{2} \right]}{-j\left[ \omega\left[ \left[ R_{w}^{2}+\left( \omega L_{w}-\frac{1}{\omega C_{0}} \right)^{2} \right]L_{p}+\left( R_{p}^{2}+\omega^{2}L_{p}^{2} \right)L_{w} \right]-\frac{\left( R_{p}^{2}+\omega^{2}L_{p}^{2} \right)}{\omega C_{0}} \right]+\left[ R_{w}^{2}+\left( \omega L_{w}-\frac{1}{\omega C_{0}} \right)^{2} \right]R_{p}+\left( R_{p}^{2}+\omega^{2}L_{p}^{2} \right)R_{w}}$ | (8) |
| $B=\omega\left[ \left[ R_{w}^{2}+\left( \omega L_{w}-\frac{1}{\omega C_{0}} \right)^{2} \right]L_{p}+\left( R_{p}^{2}+\omega^{2}L_{p}^{2} \right)L_{w} \right]-\frac{\left( R_{p}^{2}+\omega^{2}L_{p}^{2} \right)}{\omega C_{0}}=0$ | (9) |

we define *B* to indicate the imaginary part of *Z*, then, when *B* equals to 0, we can realize minimum value of *I*_2_ and maximum voltage supply. In our measurements, the impedances of DPLUS before adding the inductor at 0.811, 1.346, 1.53, 1.706 MHz are around 20, 12, 10, 8 Ω, *L*_w_ and *R*_w_ are around 77 nH and 30 mΩ, *R*_p_ is around 65 mΩ, *C*_0_ is around 9, 8.65, 8.3, and 9.7 nF at 0.811, 1.346, 1.53, 1.706 MHz, respectively. By using Supplementary Equation (9), we designed two inductors, one with 1.475 µH for 1.346, 1.53, 1.706 MHz and the other with 3.44 µH for 0.811 MHz. The impedance with designed inductor at these frequencies are 53, 28, 18, 13 Ω, respectively.


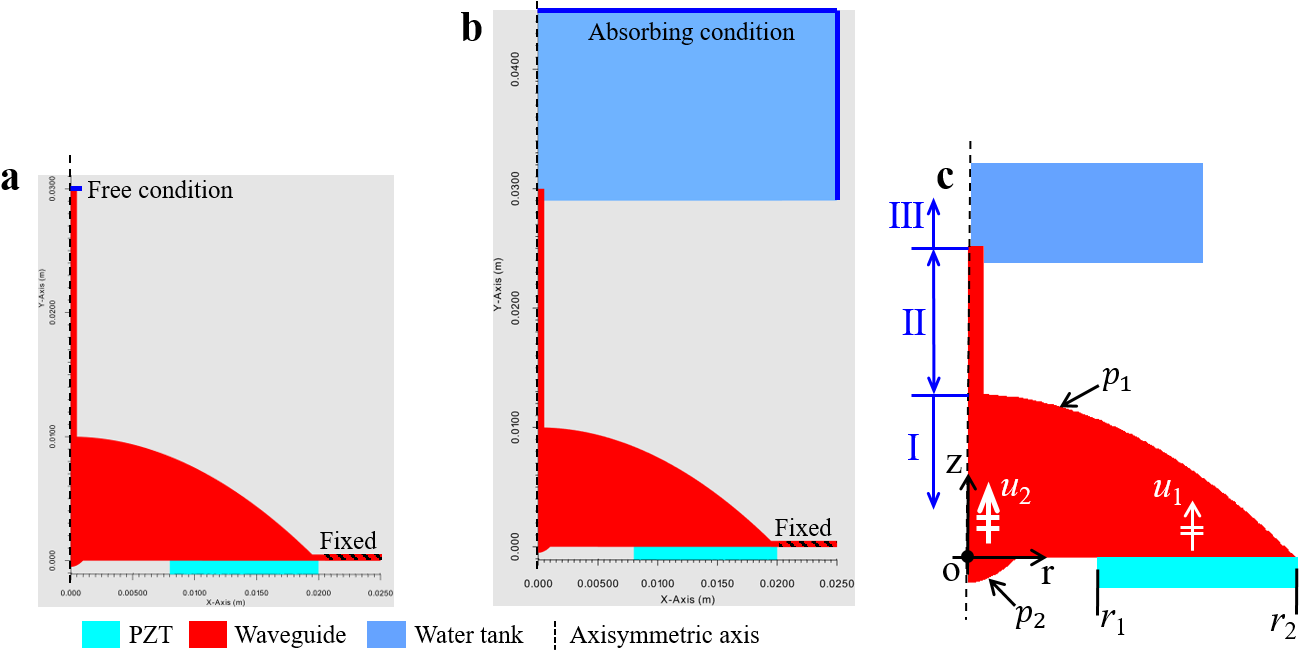


**Supplementary Figure 6: Models of the waveguide.** Simulation models (a) without water tank and (b) with water tank in PZFlex to obtain the vibration velocity at the waveguide tip and acoustic pressure in water. (c) Modelling of the waveguide. The wave propagation in the waveguide is divided into three regions.


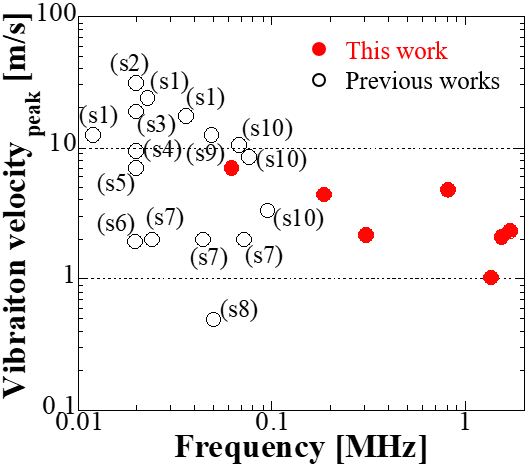


**Supplementary Figure 7: Vibration velocity comparison with conventional high-power transducers for tissue destruction.** (36), (38), (39) refer to [36], [38], [39] in the paper, s1 to s7 refer to Supplementary references [1]-[7].


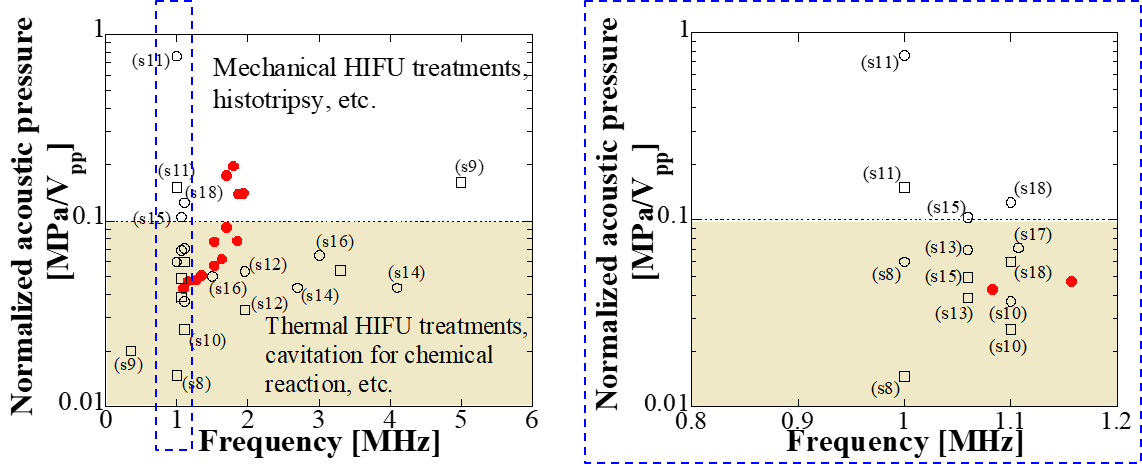


**Supplementary Figure 8: Acoustic pressure comparison with conventional HIFU transducers.** s12 to s22 refer to Supplementary references [8]-[18]. Black circles represent the peak positive pressure, black squares indicate the peak negative pressure. For our waveguide, the measured acoustic pressure was at 10 V_pp_ but the compared normalized pressure was calculated to the maximum applied voltage according to the vibration velocity values. The calculation formula is *v*_2_/*v*_1_=*p*_2_/*p*_1_, where *v*_2_ and *v*_1_ are the vibration velocities under voltage *V*_1_, *V*_2_, *V*_1_ is 10 V_pp_ and *V*_2_ is the maximum applied voltage, *p*_2_ and *p*_1_are the corresponding peak positive acoustic pressure.

**References**

1. Rosenschein, U. *et al.* Experimental ultrasonic angioplasty: disruption of atherosclerotic plaques and thrombi in vitro and arterial recanalization in vivo. *J. Am. Coll. Cardiol.* **15**, 711-717 (1990).
2. Siegel, R. J., Fishbein, M. C., Forrester, J., Moore, K. & Donmichael, T. A. Ultrasonic plaque ablation: a new method for recanalization of partially or totally occluded arteries. *Circulation* **78**, 1443-1448 (1989).
3. Ariani, M. *et al.* Dissolution of peripheral arterial thrombi by ultrasound. *Circulation* **84**, 1680-1688 (1991).
4. Inui, T., Kurashina, Y., Imashiro, C. & Takemura, K. Method of localized removal of cells using a bolt-clamped Langevin transducer with an ultrasonic horn. *Eng Life Sci.* **19**, 575-583 (2019).
5. Mathieson, A. *et al.* Ultrasonic needles for bone biopsy. *IEEE Trans. Ultrason. Ferroelectr. Freq. Control* **64**, 433-440 (2017).
6. Khalaji, I., Hadavand, M., Asadian, A., Patel, R. V. & Naish, M. D. in *2013 IEEE/RSJ International Conference on Intelligent Robots & Systems*, 4099-4104 (Tokyo, Japan, 2013).
7. Lockhart, R. *et al.* Silicon micromachined ultrasonic scalpel for the dissection and coagulation of tissue. *Biomed. Microdevices* **17**, 77 (2015).
8. Zhou, Y. Acoustic power measurement of high-intensity focused ultrasound transducer using a pressure sensor. *Med. Eng. Phys.* **37**, 335-340 (2015).
9. Lin, K. W., Hall, T. L., Xu, Z. & Cain, C. A. Histotripsy lesion formation using an ultrasound imaging probe enabled by a low-frequency pump transducer. *Ultrasound Med. Biol.* **41**, 2148-2160 (2015).
10. Martin, E. & Treeby, B. Investigation of the repeatability and reproducibility of hydrophone measurements of medical ultrasound fields. *J. Acoust. Soc. Am.* **145**, 1270-1282 (2019).
11. Rosnitskiy, P. B., Yuldashev, P. V., Sapozhnikov, O. A., Maxwell, A. D. & Khokhlova, V. A. Design of hifu transducers for generating specified nonlinear ultrasound fields. *IEEE Trans. Ultrason. Ferroelectr. Freq. Control* **64**, 374-390 (2016).
12. Brayman, A. A. *et al.* Inactivation of Planktonic Escherichia coli by Focused 2-MHz Ultrasound. *Ultrasound Med Biol.* **43**, 1476-1485 (2017).
13. Bessonova, O. V. & Wilkens, V. Membrane hydrophone measurement and numerical simulation of HIFU fields up to developed shock regimes. *IEEE Trans. Ultrason. Ferroelectr. Freq. Control* **60**, 290-300 (2013).
14. Jeong, J. S., Cannata, J. M. & Shung, K. K. Dual-focus therapeutic ultrasound transducer for production of broad tissue lesions. *Ultrasound Med. Biol.* **36**, 1836-1848 (2010).
15. Haller, J., Wilkens, V. & Shaw, A. Determination of acoustic cavitation probabilities and thresholds using a single focusing transducer to induce and detect acoustic cavitation events: I. method and terminology. *Ultrasound Med Biol.* **44**, 377-396 (2018).
16. Ma, J., Guo, S., Wu, D., Geng, X. & Jiang, X. Design, fabrication, and characterization of a single-aperture 1.5-Mhz/3-Mhz dual-frequency HIFU transducer. *IEEE Trans. Ultrason. Ferroelectr. Freq. Control* **60**, 1519-1529 (2013).
17. Hariharan, P. *et al.* Characterization of high intensity focused ultrasound transducers using acoustic streaming. *J. Acoust. Soc. Am.* **123**, 1706-1719 (2008).
18. Martin, E., Zhang, E. Z., Guggenheim, J. A., Beard, P. C. & Treeby, B. E. Rapid Spatial Mapping of Focused Ultrasound Fields Using a Planar Fabry-Pérot Sensor. *IEEE Trans. Ultrason. Ferroelectr. Freq. Control* **64**, 1711-1722 (2017).
